# Monkeys who experience more feeding competition utilize social information to learn foraging skills faster

**DOI:** 10.1038/s41598-023-37536-9

**Published:** 2023-07-19

**Authors:** T. Jean M. Arseneau-Robar, Karyn A. Anderson, Pascale Sicotte, Julie A. Teichroeb

**Affiliations:** 1 Department of Anthropology, University of Toronto Scarborough, Toronto, ON Canada; 2 Department of Anthropology, University of Toronto, Toronto, ON Canada; 3 Department of Biology, Concordia University, Montreal, QC Canada

**Keywords:** Behavioural ecology, Feeding behaviour, Learning and memory, Social behaviour

## Abstract

Animals must learn foraging skills to successfully survive and reproduce but the sources of interindividual variation in learning are poorly understood. For example, there is little consensus on the role motivation plays, even though it is a key factor impacting learning outcomes in humans. Here, we conduct a field experiment on a wild primate to investigate whether an individual’s vulnerability to feeding competition impacts their motivation to learn a beneficial foraging technique. We provided a group of monkeys with a food reward (i.e., a half banana) that needed to be retrieved from a box. The monkeys discovered an efficient technique that consistently allowed them to retrieve the banana quickly, decreasing the risk of food loss to competitors. We found that individuals who frequently experienced feeding competition learned this efficient technique significantly faster than individuals who rarely foraged in the presence of a dominant competitor. They appeared to use social learning to learn faster as they were more attentive to the handling techniques others used and improved their foraging skills after opportunities to observe a skilled demonstrator. These findings support that an individual’s vulnerability to feeding competition impacts their motivation to learn foraging skills that reduce food loss to competitors.

## Introduction

To survive, animals must overcome a multitude of ecological and social challenges. Individuals may need to learn to forage optimally, find or construct shelter, avoid predation, attract mates, form enduring social bonds, and outcompete conspecifics^1–3^. The need to acquire such a diverse skillset is thought to create selective pressure for the evolution of long juvenile periods, and this trait is exaggerated in many social species like primates, elephants and cetaceans, where around a fifth of the lifespan is spent in childhood^1,2^. While many skills can be learned individually, doing so is often risky and costly, as individuals may need to explore their environment, innovate new solutions, and improve their skills through practice. Conversely, individuals can learn from observing or interacting with others (referred to as social learning) and acquire information from knowledgeable conspecifics^4^. As a result, social learning typically allows individuals to learn faster^5–8^ and achieve a higher level of competency^9,10^.

The past three decades have generated a wealth of knowledge on learning in non-human animals, with researchers increasingly working on wild populations. By studying animals in natural settings, where they are subject to the extensive array of selective pressures that have shaped their evolutionary trajectory, we have broadened our knowledge on the skills animals learn, and who they learn from^11–20^. However, we understand comparatively little about the sources of variation in learning success and efficiency among individuals. Inter- and intraindividual variation in learning may arise from differences in personality, aptitude, or an individual’s motivation to learn^21^. Given that motivation is recognized as a key factor influencing learning outcomes in humans^22,23^, greater attention to its impact in other species is warranted. Motivation is an internal process that energizes, directs, and sustains behaviour. Whether consciously or unconsciously, individuals weigh the perceived value (i.e., utility) gained from completing a task against the effort required to do so^22,23^. Thus, individuals should be motivated to learn a new skill if they perceive the benefits to outweigh the opportunity costs and cognitive effort of learning. Importantly, because it is a psychological construct, motivation cannot be observed directly. Instead, it must be inferred by measuring an individual’s cognitive, affective, behavioural or physiological responses, or by asking test subjects how motivated they felt to learn^24^. Given that many of these measures cannot be obtained in wild animals, who lack our advanced language capabilities, task performance metrics (e.g., speed, success or persistence) are often the best measures we have to infer motivation^25–27^.

How motivated individuals are to learn a new foraging skill may depend on their (a) current competency, (b) hunger level, (c) the impact that food resources have on their fitness, or (d) their competitive ability. For example, it is thought that juveniles may be motivated to learn foraging skills because of their asymmetry in knowledge compared to adults^28,29^. Those who are hungry, in poor condition or experiencing food scarcity are expected to value skills that increase food access more than those who are not^30,31^. It has been posited that females may be more motivated to acquire foraging skills than males because their high levels of investment in offspring means that female fitness is typically limited by access to food resources^32,33^. Lastly, individuals who are small, have poor fighting skills, or have a low dominance rank may be more motivated to learn new foraging skills than individuals who are able to win access to valuable food resources via contest competition^34–36^.

While many studies have attempted to test whether poor competitive ability motivates individuals to learn new foraging skills, there is little consensus in the literature on whether ‘necessity is the mother of invention’^34–36^. In some cases, individuals who are thought to be at a competitive disadvantage can be more likely to innovate a new foraging skill, more persistent in their attempts to innovate, or solve a foraging problem faster^36–44^. However, numerous studies have failed to find a correlation between age, size or dominance rank and innovation persistence, speed or success^45–49^. Some authors have suggested that individuals who are motivated to learn may refrain from innovating because they want to avoid conflict with high-ranking audience members or are sensitive to the risk of theft^50–52^. Others have concluded that high-ranking individuals have a greater capacity to innovate^35,48^. However, it is also likely that in many cases, the rough proxies that are used to measure individual variation in the perceived utility of learning the foraging skill, do not accurately reflect how motivated individuals felt in the current conditions. The inappropriateness of rough proxies for motivation has also been suggested by Thornton and Samson^44^ as there is a similar lack of consensus on the impact that hunger state has on the propensity to innovate^35,51^. These conflicting results make it difficult to draw any firm conclusions on the role that motivation plays in driving learning in non-human animals, or the factors that determine how motivated individuals feel to learn.

**Figure 1:**
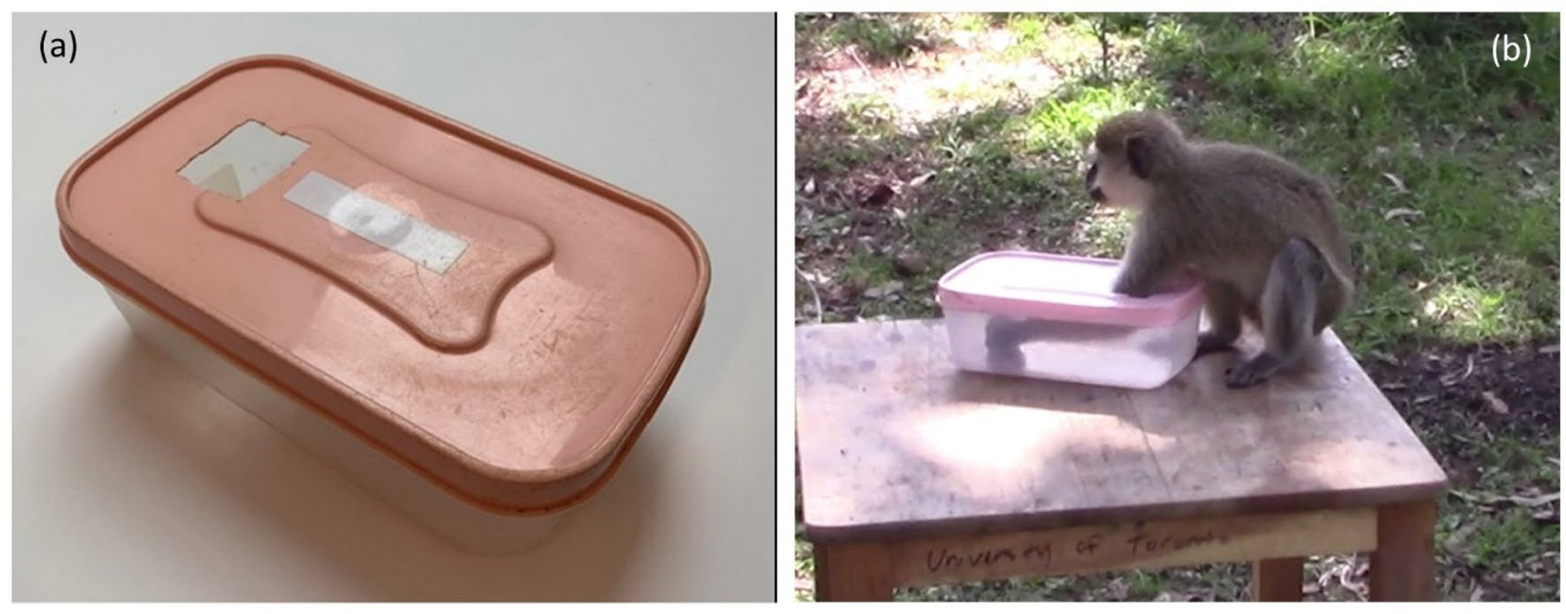
(**a**) The food box in which the half-banana was placed, and (**b**) a vervet monkey retrieving the food reward using the no-manipulation reach-in technique. With this technique, they refrained from handling the box (i.e., left it sitting on the table) and instead inserted their whole arm in so they could grab the half-banana at the far end, and pull it out.

The aim of this study was to investigate whether competitive ability impacts an individual’s motivation to learn a more-efficient solution to a foraging problem. We conducted a field experiment on a wild group of vervet monkeys (*Chlorocebus pygerythrus*), a species which forms linear dominance hierarchies^53^ that affect their ability to monopolize food resources^54–56^. We presented the monkeys with a valuable food reward (i.e., a half banana), which needed to be retrieved from a clear plastic box with a hole on one side of the opaque lid (Fig. 1a). We did not train a demonstrator, but rather allowed the monkeys to innovate their own solutions. The monkeys initially developed handling techniques that focused on manipulating the box until the banana fell out, or was near the hole and could be grabbed by inserting only their hand into the box (ESM videos 1, 2). Subsequently, however, they invented an alternative technique in which they refrained from handling the box, and instead reached into the far end, grabbed the banana, and pulled it out. This “no-manipulation reach-in” technique (Fig. 1b; ESM videos 3, 4) was more efficient as handlers did not spend time rolling, tipping, or shaking the box (ESM Fig. 1). While much of the experimental work on learning in wild animals investigates the learning of new skills (by presenting them with a novel apparatus to solve), our investigation into the role motivation plays in improving a skill is biologically relevant as many of the skills that animals learn in the wild are improvements or refinements to existing techniques. For example, moss sponging in chimpanzees (*Pan troglodytes*) is a more efficient drinking technique than leaf sponging^57^, and the hooked tools built by New Caledonian crows (*Corvus moneduloides*) are more effective when extractive foraging than unmodified tools^58^. In the experiment we conduct here, learning the no-manipulation reach-in was beneficial because being able to reliably retrieve the banana within a few seconds allowed individuals to obtain the highly-valued food reward (i.e. the banana) before dominant audience members could arrive at the experiment and displace them^55^.

In line with the ‘necessity is the mother of invention’ hypothesis^34–36^, we expected that individuals who experienced more feeding competition would be more likely to learn the no-manipulation reach-in technique and learn it faster. However, instead of using rough proxies of individual competitive ability (e.g., age, size or rank), we explored how individual group members experienced feeding competition when trying to access the food resources provided in this experiment. We did this because, while their propensity to form linear dominance hierarchies means that rank is likely a key factor impacting the amount of feeding competition individuals experience, other factors likely influence motivation as well. For example, it is possible that individuals who are frequently tolerated by dominants around food resources, are willing forage away from the safety of the group where contest competition is less likely to arise (i.e., be a producer), or are skilled at sneaking or stealing food from competitors^54–56^ may feel less motivated to learn as they can access food resources through alternative strategies. This approach allowed us to choose a more precise measure of individual vulnerability to contest competition, which we expected would index motivation to learn this particular foraging skill (i.e., the no-manipulation reach-in technique) more accurately than dominance rank.

We also hypothesized that individuals who were highly motivated to learn efficient foraging techniques would rely on social learning to gain competency quickly, because it has been demonstrated in other studies to be a more rapid learning mode than individual (i.e., trial-and-error) learning^5–8^. Therefore, we expected that individuals who experienced high levels of feeding competition would both seek out social information (i.e., attend to the handling techniques of others), and improve their skills after having opportunities to learn socially. Conversely, we predicted that individuals who experience little contest competition would be more likely to ignore social information. Notably, this lack of investment in social learning by high-ranking individuals has been observed in chimpanzees^59^.

## Methods

This research was conducted from January to April 2019, at a field site near Lake Nabugabo, Uganda (0°22′–12° S, 31°54′ E). Study subjects were the members of one habituated group of wild vervet monkeys (*Chlorocebus pygerythrus*), which contained 4 to 7 adult males at any given time, 10 adult females, 3 subadult males (i.e., ≥ 3 years old and still residing in their natal group), 5 subadult females (≥ 3 years old and nulliparous), and 16–19 juveniles (≤ 2 years old). However, juveniles were rarely able to gain access to the food box and so were not considered in this study.

We conducted a foraging experiment that involved providing the monkeys with access to a multi-destination array comprised of five platforms, one of which was baited with a valuable food reward (i.e., a half banana) that needed to be retrieved from a food box. The experimental methodology was approved by the Uganda Wildlife Authority, the Uganda National Council for Science and Technology, and the University of Toronto Animal Care Committee. The methods were carried out in accordance with these protocols and the ARRIVE guidelines. We had two goals with this experiment; the first was to examine how the risk of feeding competition impacted foraging decisions^55^, and the second was to investigate interindividual variation in learning (this study). In each experimental trial, an observer baited the platforms, recorded the identity of the monkeys who obtained food rewards, the composition of the audience (within 100 m) and their distance from the experiment, and any social interactions that were observed. The observer also video recorded each trial so that the techniques that were used to retrieve the banana from the food box could be coded in detail, and the time spent handling measured. We did not restrict access to the food box, meaning each group member could obtain the food reward if they could handle the box without being displaced by a competitor, and successfully retrieve the banana. This task was relatively easy as all group members quickly learned to get the banana out, however, there was a lot of variation in handling times. The most efficient handling technique the monkeys innovated did not actually involve manipulating the box. Instead, the handing individual inserted their arm up to the elbow so they could reach the banana at the far end, grab it, and pull it out (i.e., no-manipulation reach-in; ESM videos 3, 4). This technique may have been relatively difficult to learn as it required the monkeys to retrieve the banana without coordinating this action visually (i.e., they could not see the banana through the opaque lid), and they had to inhibit the urge to pick the box up.

We also used the video recordings to opportunistically quantify how frequently group members attended to the handling techniques of others when present in the audience. Audience members were visible in the background of 276 trials. In each of these, we coded whether the audience member’s face was oriented towards the handling individual for at least one second while they handled the food box. We used a one-second cut-off because subordinate competitors often glanced at the handler briefly before/as they moved to feed at another platform, seemingly to check that the dominant competitor was still busy with the food box and/or ensure the dominant was going to tolerate them eating the food on another platform (ESM videos 7, 8). Conversely, when sitting and watching the handling individual, audience members typically looked at the handler for more than a second at a time (ESM videos 5, 6). This sustained attention was more likely to allow them to observe the action sequence being used by the handler, and so could provide information on the handling technique being employed. We censored out any trials in which the audience member was attempting to displace the handler, or was aggressing them, as it seemed unlikely they were focused on the handling techniques in these cases. We quantified the opportunities each individual had to learn socially by calculating the number of times they had been in the audience (i.e., within 50 m) when a group member demonstrated the no-manipulation reach-in technique, and deemed that they had recently had a social-learning opportunity if this had occurred no more than 30 min prior to them handling the box themselves. We chose 30 min because monkeys who were waiting for a chance to participate in the experiment often sat nearby (i.e., were present in the audience), but did not stay for longer than 30 min. Therefore, the observed latencies between observing the handling techniques of others and handling the food box oneself was typically less than 30 min, or one or more days later.

Each group member’s dominance rank was calculated using Elo-ratings^60–63^, using data on aggressive interactions and displacements collected around the experiment, as well as during ongoing behavioural data collection^55^ (N = 3221). Although females can out-rank males in this species^54,64,65^, all the adult males out-ranked all adult and subadult females during this study. Subadult males were subordinate to adult males but were interspersed within the female dominance hierarchy (ESM Table 1). Elo-ratings were calculated using the ‘EloRating’ package^61^ in which the known ordinal ranks at the onset of the experiment were input as “startvalues”, and the default settings were used for “k” (i.e., 100) and “normprob” (i.e., normal distribution). We used the average Elo-rating across the study period as our measure of individual dominance rank, but for each experimental trial, we used the daily Elo-ratings to score whether the individual handling the food box was in competition with a dominant or subordinate group member. Unlike ordinal ranks, higher Elo-rating values indicate that an individual was high-ranking, while low or negative Elo-rating values indicate lower dominance rank.

**Table 1:** Numbered list of models presented in this study, including the type of statistical model and its structure.

Model #	Model type	Model structure
1	GLM	Reach deeply into box yes/no ~ proportion trials with dominant competitor (scaled)
2	GLM	Attempt no-manipulation reach-in yes/no ~ proportion trials with dominant competitor (scaled)
3	GLM	Execute no-manipulation reach-in yes/no ~ proportion trials with dominant competitor (scaled)
4	GLM	Personal fastest handling time yes/no ~ proportion trials with dominant competitor (scaled)
5	GLM	Reach deeply into box yes/no ~ Elo-rating (scaled)
6	GLM	Attempt no-manipulation reach-in yes/no ~ Elo-rating (scaled)
7	GLM	Execute no-manipulation reach-in yes/no ~ Elo-rating (scaled)
8	GLM	Personal fastest handling time yes/no ~ Elo-rating (scaled)
9	GLMM	Attentive when in audience yes/no ~ proportion trials with dominant competitor + knew no-manipulation reach-in + (1|individual)
10	GLMM	Attentive when in audience yes/no ~ age-sex class + (1|individual)
11	GLMM	Adult male attempt/execute no-manipulation reach-in yes/no ~ recently observed no-manipulation reach-in demonstrated yes/no + (1|individual)
12	GLMM	Adult female or subadult attempt/execute no-manipulation reach-in yes/no ~ recently observed no-manipulation reach-in demonstrated yes/no + (1|individual)
13	GLMM	Try/execute no-manipulation reach-in if recently observed it yes/no ~ adult male yes/no + (1|individual)
14	Kendall’s Rank Correlation	Cumulative handling time needed to learn no-manipulation reach-in ~ proportion trials with dominant competitor
15	Wilcoxon Rank-Sum Test	Cumulative handling time needed to learn no-Manipulation reach-in ~ adult male yes/no

We calculated several other metrics (i.e., in addition to dominance rank) to explore each individual’s vulnerability to feeding competition to further investigate how useful dominance rank is as an index of competition experienced. Individual monkeys typically visited the experiment when travelling with the group, but some also came alone (i.e., there were no group members within 100 m/in-sight). For the former, we calculated how often each individual had a dominant competitor present when foraging with the group. We focused on dominant competitors because the monopolizability of the food box, handling time required to retrieve the banana, and linear dominance hierarchies seen in vervet monkeys, collectively meant that subordinate audience members had very little capacity to aggress a dominant or successfully steal the banana. Thus, subordinate audience members imposed few costs on dominants in this context. We also calculated how frequently each individual was displaced or aggressed when trying to access experimentally provided food resources as a more direct measure of contest competition experienced. To quantify each individual’s propensity to forage alone (i.e., escape contest competition) we calculated the number of trials each individual completed with no group members in sight. Lastly, we calculated the proportion of all trials participated in where a dominant competitor was present. This was the metric we used to index feeding competition experienced when running the rest of the analyses as this measure reflected the amount of contest competition individuals experienced when group-foraging (which was largely dependent on their dominance rank), as well as their willingness to forage away from the safety of the group to escape feeding competition.

### Statistical analyses

All data supporting this manuscript are included in the Electronic Supplementary Materials. We first explored how individuals experienced feeding competition when participating in this experiment. Because the data did not meet the assumptions of normality, we used Kendall’s rank correlations to investigate the relationship between dominance rank and (1) the proportion group-foraging trials in which the individual handling the food box was in competition with a dominant competitor, (2) the proportion of group-foraging trials in which they were aggressed/displaced by a dominant competitor, and (3) the number of trials in which they foraged solitarily with no group members in sight. We also used a Kruskal–Wallis test, with a Holm-Bonferroni adjustment for multiple comparisons^66^, to examine differences in behaviour among the age-sex classes.

We used four models to assess how the amount of feeding competition experienced (i.e., the proportion of all experimental trials that each individual had a dominant competitor present) impacted the speed with which individuals gained proficient handling skills. The four benchmarks we considered were the number of handling events each individual took to (1) reach deeply into the food box (rather than manipulate the box until the banana was easy to grab)(model 1, Table 1), (2) attempt a no-manipulation reach-in (i.e., inhibit the urge to manipulate the food box)(model 2, Table 1), (3) succeed in a no-manipulation reach-in for the first time (model 3, Table 1), and (4) achieve their personal fastest handling time (model 4, Table 1). Because the response variable in all these models were counts, and all models suffered from over-dispersion (assessed using the 'DHARMa' package, version 0.3.3.0)^67^, we used negative binomial generalized linear models (GLMs)^68^, which effectively mitigated this issue. We also scaled predictor variables, using the ‘scale()’ function in R, to improve model performance. We repeated this process using average rank as the predictor variable in these four, negative binomial GLMs (models 4 to 8, Table 1), and used AIC to compare how well dominance rank (i.e., a rough proxy for competitive ability) and the proportion of trials with a dominant competitor present (i.e., a more precise measure of competition experienced in this foraging task) each explained variation in learning.

To understand how some individuals learned faster than others, we examined (1) the conditions in which audience members were attentive to the handling techniques other’s used, and (2) the learning experiences individuals had before they successfully executed the no-manipulation reach-in technique. Using a generalized linear mixed model (GLMM) to control for repeated observations of individuals^69,70^, we tested whether the amount of feeding competition experienced (i.e., the proportion of trials with a dominant competitor) and the audience members own skill level (i.e., whether they had learned the no-manipulation reach-in technique yet) impacted the propensity to attend to the handling techniques of others (model 9, Table 1). Similarly, we used a GLMM to examine age-sex differences in attention (i.e., whether they attended to the handling techniques used by the focal when in the audience), by including age-sex class as the predictor variable and setting adult males to the reference category that the other age-sex classes were compared to (model 10, Table 1).

**Figure 2:**
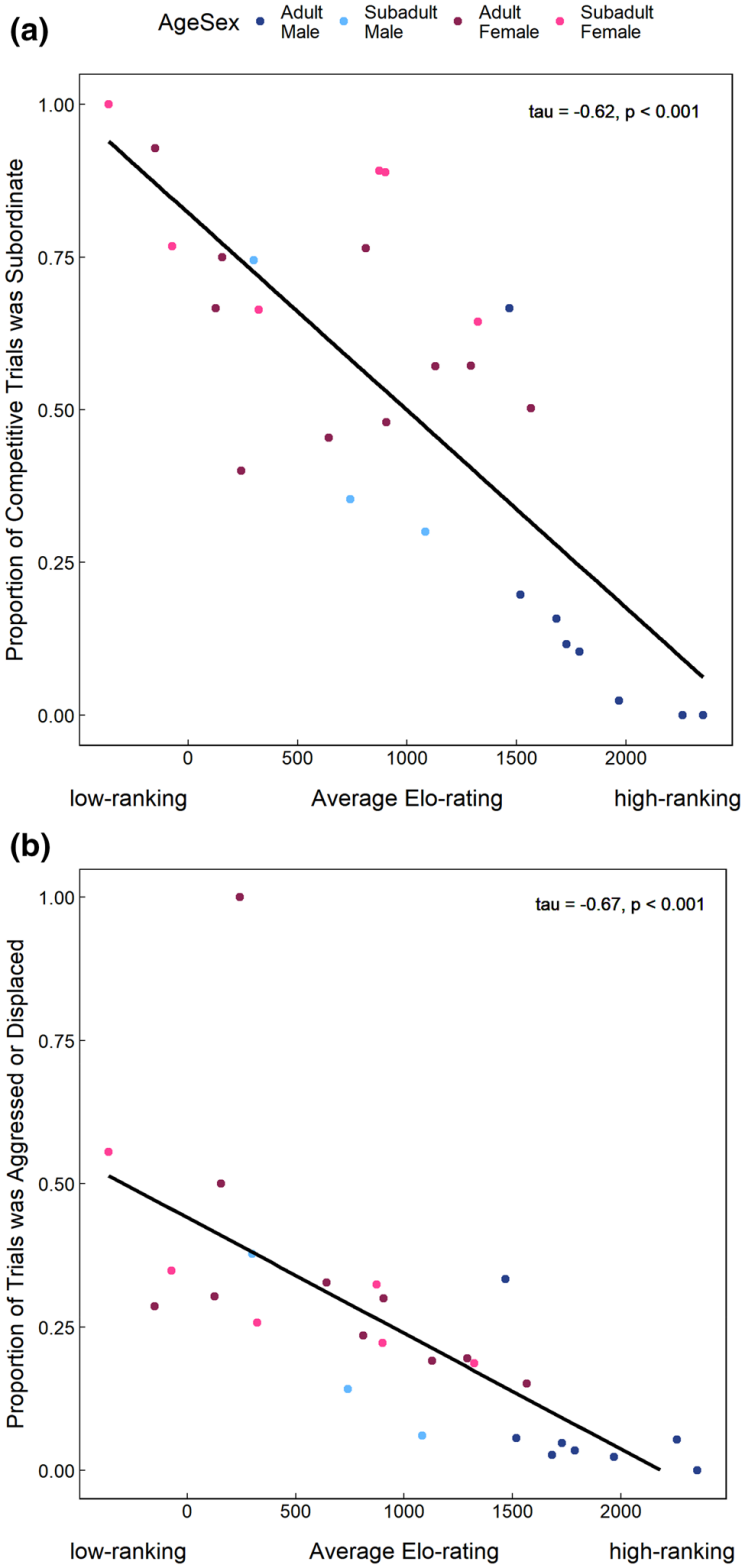
The relationship between dominance rank (i.e., average Elo-rating throughout the study period) and the proportion of competitive trials in which individuals (**a**) were the subordinate of the two competitors, and (**b**) were aggressed or displaced when they attempted to access food resources. Higher Elo-ratings indicate higher dominance rank, whereas low or negative values indicate a low dominance rank.

To investigate how learning experiences impacted learning success, we examined all handling events that each individual obtained before successfully executing the no-manipulation reach-in technique. Here, we lacked the sample size to test for an interaction between the amount of feeding competition experienced, and whether the individual had had a recent opportunity to observe the no-manipulation reach-in technique. So, we instead created two GLMMs, one in which the focal monkey was an adult male and one in which they were one of the other age-sex classes (models 11 and 12, Table 1). While we advocate against using rough proxies for competitive ability, we begin our analyses by investigating how individuals experience feeding competition. This highlighted that adult males were rarely at risk of losing the valued food reward to a dominant competitor if they failed to extract it from the box quickly enough. Consequently, adult males experienced feeding competition very differently from the other age-sex classes (e.g., Fig. 2). In each of these two GLMMs we tested whether individuals were more likely to attempt, or successfully execute the no-manipulation reach-in technique if they had recently observed a group member demonstrate it (models 11 and 12, Table 1). If individuals are using social learning, we would expect them to be most likely to try/use the no-manipulation reach-in after observing another monkey demonstrate it (i.e., see-it, do-it)^13,17,71^. We were also wary of the possibility that adult males lacked opportunities to learn socially because they were able to monopolize the food box for long periods. Therefore, we examined a subset of data in which individuals had had the opportunity to learn from a skilled demonstrator, as well as the opportunity to handle the food box in the near future (i.e., within 30 min). Using these data, we built a GLMM to test whether adult males were less likely than the other age-sex classes to try or successfully execute the no-manipulation reach-in themselves if they had had a recent social learning opportunity (model 13, Table 1).

Lastly, we used a Kendall’s rank correlation, as well as a Wilcoxon rank-sum test, to investigate the role that individual learning played acquiring proficiency. Here, we tested how the amount of feeding competition experienced (model 14, Table 1), and whether an individual was an adult male or not (model 15, Table 1), related to the cumulative handling time needed to successfully execute the no-manipulation reach-in technique. All statistical analyses were conducted in R (version 3.6)^72^. Negative binomial GLMs were implemented using the ‘MASS’ package (version 7.3–51.4)^73^ and GLMMs were built using the ‘lme4’ package^70^. We used the 'DHARMa' package (version 0.3.3.0)^67^ to ensure models did not suffer from overdispersion. We used the likelihood ratio test to assess the significance of predictor variables in each model^66,69^, and when predictor variables had more than two factor levels we used p-values estimated using the Wald test to assess the significance of each dummy variable. For example, when using GLMMs to compare the behaviour of age-sex classes, we set adult males to the reference category and compared the other age-sex classes to them. We examined model fit in the GLMMs by comparing the full model (model with all fixed effects included) to the null model (model with random effects only) using likelihood ratio tests and using residual diagnostics. All GLMM models with significant fixed effects performed significantly better than the null model (all *χ*^2^ ≥ 3.98, all *p* < 0.040).

## Results

In total, we observed the handling techniques the monkeys used to retrieve the banana from the food box in 1648 trials. Every adult and subadult in the group was able to handle the box at least once, but the number of handling opportunities that each study participant obtained varied from 1 to 264. The amount of time it took the monkeys to retrieve the banana from the food box also varied considerably, with the fastest handling time observed being 1 s and the longest being 69 s (x̄ = 6.7 s). All group members improved their skill level with experience (ESM Fig. 2), as we observed significant improvements in handling time between each individual’s first handling event and their personal best (Binomial test: *N* = 24 monkeys had more than one handling opportunity, *mu* = 0, *p* < 0.001). The majority of monkeys also learned to retrieve the banana from the food box using the no-manipulation reach-in technique. Of the 27 study participants, only one subadult female, who was the lowest ranking participant in the experiment, failed to learn it. Given that she was only able to handle the box once, she likely did not obtain sufficient opportunities to learn. We observed significant variation in the speed with which individuals learned, with individuals needing anywhere from one to 44 handling opportunities to successfully execute the no-manipulation reach-in technique.

Instead of assuming there was a strong link between dominance rank and competitive ability, we explored how individuals experienced feeding competition to infer their motivation to learn the no-manipulation reach-in technique. As would be expected, we found that when they were foraging with the group, low-ranking individuals were significantly more likely to have a dominant competitor present at the experiment than their high-ranking group mates (Fig. 2a). Consequently, low-ranking individuals were also more likely to experience contest competition as they were more likely to be aggressed or displaced by a competitor (Fig. 2b). Notably, adult males out-ranked all other group members during this study (ESM Table 1), and almost never co-fed at the platforms with another adult male. As a result, they were rarely in competition with a dominant competitor, aggressed or displaced from the experiment platforms (Fig. 2a,b).

**Figure 3:**
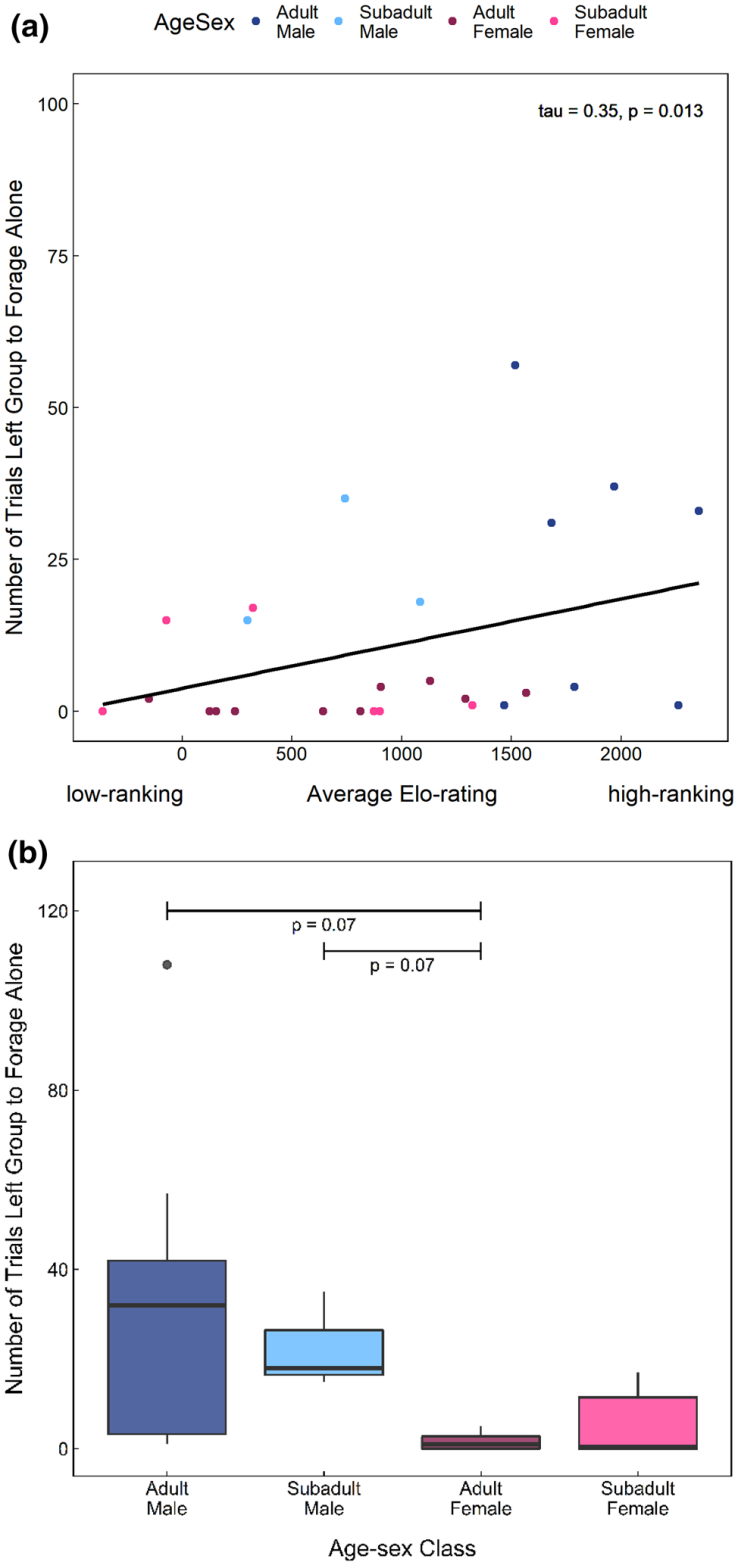
Propensity for individuals to leave the group and visit the experiment site in solitude (i.e., no group members were within 100 m) relative to their (**a**) dominance rank and (**b**) age-sex class. Higher Elo-ratings indicate higher dominance rank, whereas low or negative values indicate a low dominance rank.

Importantly, individuals could also avoid contest competition by leaving their group and visiting the experiment site alone. We found that high-ranking individuals displayed a significantly greater propensity to forage solitarily (Fig. 3a), however, this rank effect was likely also impacted by sex differences in willingness to leave the safety of the group, as both adult and subadult males showed a greater tendency to forage solitarily than adult females (Fig. 3b). They also stayed away from the group for longer. While subadult and adult males were observed to remain, alone, at the experiment site for up to 30 min, adult females never stayed alone for more than 4 min.

To ensure our measure of competitive ability reflected both the levels of feeding competition arising from an individual’s position in the dominance hierarchy, and their ability to escape competition by foraging solitarily, we calculated the proportion of all foraging trials participated in that individuals had a dominant competitor present. We then assessed how competition experienced impacted learning efficiency using four milestones. We found that individuals who frequently foraged in the presence of a dominant competitor, learned the no-manipulation reach-in technique significantly faster than those who did not. They took fewer trials to start reaching deeply into the food box (i.e., reach-in up to their elbow) (Model 1, GLM: *N* = 26, *β* = −1.25, *SE* = 0.55, *χ*^2^ = 5.11, *p* = 0.024), to attempt the no-manipulation reach-in (i.e., inhibit the urge to manipulate the food box) (Model 2, GLM: *N* = 26, *β* = −2.40, *SE* = 0.65, *χ*^2^ = 14.24, *p* < 0.001), to successfully execute this technique (Model 3, GLM: *N* = 26, *β* = −2.68, *SE* = 0.62, *χ*^2^ = 20.28, *p* < 0.001), and to achieve their personal fastest handling time (Model 4, GLM: *N* = 26, *β* = −2.19, *SE* = 0.65, *χ*^2^ = 11.72, *p* < 0.001). Notably, the proportion of trials with a dominant competitor present explained more of the variation in individual learning speed than dominance rank did (Table 2, all delta AICc values > 2). While this rough proxy for competitive ability did a good job of explaining variation in learning speed according to some milestones (Model 6, GLM, attempting the no-manipulation reach-in: *N* = 26, *β* = 0.43, *SE* = 0.20, *χ*^2^ = 4.38, *p* = 0.036; Model 7, GLM: executing it successfully: *N* = 26, *β* = 0.51, *SE* = 0.20, *χ*^2^ = 6.65, *p* = 0.010), it did a poor job of explaining how long it took individuals to start reaching deeply into food box (Model 6, GLM: *N* = 26, *β* = 0.20, *SE* = 0.16, *χ*^2^ = 1.57, *p* = 0.211), or to achieve their personal fastest handling time (Model 8, GLM: *N* = 26, *β* = 0.31, *SE* = 0.21, *χ*^2^ = 3.29, *p* = 0.069).

**Table 2:** Comparison of model performance when using dominance rank (i.e., a rough proxy) versus a more precise measure of competition experienced to explain individual variation in learning speed.

	df	AICc	Delta AICc
Reach arm in deeply			
Competition experienced	3	103.8	0.0
Rank	3	107.1	3.3
Attempt no-manipulation reach-in			
Competition experienced	3	131.8	0.0
Rank	3	139.7	7.8
Execute no-manipulation reach-in			
Competition experienced	3	135.8	0.0
Rank	3	145.5	9.7
Achieve personal fastest handling time			
Competition experienced	3	198.6	0.0
Rank	3	205.4	6.8

Four learning milestones were considered: the number of trials before an individual reached their arm into the food box up to the elbow, attempted the no-manipulation reach-in technique, executed it successfully, and achieved their personal fastest handling time.

To understand how some individuals learned faster than others, we examined patterns of attention and the learning experiences individuals had before they successfully executed the no-manipulation reach-in technique. We found that individuals who were not yet skilled handlers (i.e., had not yet learned the no-manipulation reach-in technique) were significantly more likely to attend to the handling techniques of others (Model 9, GLMM: *N* = 276, *β* = −1.36, *SE* = 0.41, *χ*^2^ = 11.47, *p* < 0.001), as were individuals who were frequently in competition with dominant competitors (Model 9, GLMM: *N* = 276, *β* = 2.39, *SE* = 0.85, *χ*^2^ = 6.60, *p* = 0.010). In particular, the highest-ranking group members, adult males, rarely paid attention to the handling techniques used by others (attentive in 6% of cases). As a result, they were significantly less attentive than adult females (Model 10, GLMM: *N* = 276, *β* = 1.87, *SE* = 0.66, *z* = 2.83, *p* = 0.005), subadult females (Model 10, GLMM: *N* = 276, *β* = 1.68, *SE* = 0.74, *z* = 2.27, *p* = 0.023) and subadult males (Model 10, GLMM: *N* = 276, *β* = 1.62, *SE* = 0.81, *z* = 1.99, *p* = 0.046), who watched others handle the food box in approximately 25% of the trials in which they were in the audience.

The learning experiences individuals had before they learned the most efficient foraging technique showed that the age-sex classes that were most vulnerable to feeding competition (i.e., adult females, subadult females and subadult males) were more likely to attempt, or successfully execute, the no-manipulation reach-in technique if they had recently had the opportunity to learn socially. That is to say, they were more likely to show improvements in their handling technique if they had seen a group member demonstrate the no-manipulation reach-in technique within the past 30 min (Model 12, GLMM: *N* = 55, *β* = 1.12, *SE* = 0.57, *χ*^2^ = 3.98, *p* = 0.046). Conversely, for adult males, improvements in handling technique were not dependent on the occurrence of recent social learning opportunities (Model 11, GLMM: *N* = 87, *β* = 0.43, *SE* = 0.85, *χ*^2^ = 0.24, *p* = 0.625). However, this may have been because they rarely obtained opportunities to observe others. To control for differences in opportunity, we examined only the trials in which the individual handling the food box had recently observed a group member demonstrate the no-manipulation reach-in technique. Adult males were significantly less likely to attempt to copy the technique in their next handling event than the other age-sex classes (Model 13, GLMM: *N* = 43, *β* = −1.69, *SE* = 0.75, *χ*^2^ = 4.70, *p* = 0.024). Thus, high-ranking adult males ignored valuable social information when it was available. Furthermore, adult males tended to have higher cumulative handling times than the other age-sex classes by the time they successfully executed the no-manipulation reach-in technique (Model 15, Wilcoxon rank-sum test: *N* = 26, *W* = 103.5, *p* = 0.084). The same pattern was observed when looking at how competition experienced impacted the cumulative handling time to learn the no-manipulation reach-in technique as those who rarely had dominant competitors present needed more experience with the food box to learn (Model 14, Kendall’s Rank Correlation: *N* = 26, *tau* = −0.34, *z* = −2.38, *p* = 0.017). These findings suggest that adult males, and other high-ranking individuals may be more likely to learn through trial-and-error. Note that their lack of interest was not because they had already learned an alternative handling technique that worked reasonably well, as adult males frequently needed long handling times to retrieve the banana until they learned the no-manipulation reach-in technique (ESM Fig. 3).

## Discussion

The aim of this study was to investigate whether competitive ability impacts motivation to learn a foraging skill that can improve access to valuable food resources. Although almost all study participants successfully learned the most efficient technique for retrieving a food reward from a box (i.e., the no-manipulation reach-in), individuals who experienced high levels of feeding competition (i.e., frequently foraged in the presence of a dominant competitor) learned this technique significantly faster. They required fewer handling opportunities to both attempt and succeed in executing the no-manipulation reach-in technique, and to achieve their personal fastest handling time. These findings suggest that it was individuals who often had to worry about dominants displacing them from food patches that were most motivated to learn a skill that mitigated the costs of contest competition. Our findings further suggest that they used social learning to gain proficiency quickly. The age-sex classes that experienced the most contest competition (i.e., were most likely to be aggressed or displaced from food patches) were more attentive to the handling techniques others employed and were more likely to improve their handling technique after observing a skilled demonstrator. Conversely, adult males, who out-ranked all the other age-sex classes and experienced very little contest competition, were more likely to ignore valuable social information, and less likely to improve their technique after social learning opportunities. The lack of evidence for social learning in adult males, combined with their tendency to need lots of time to practice their handling techniques, suggests that adult males are more likely to improve their foraging skills through trial-and-error.

Our study is unique in that we thoroughly explore how individuals experienced competition to understand how motivated they may have felt to learn this specific foraging technique, given the current social and ecological context. All the monkeys that participated in the experiment quickly discovered techniques for retrieving the banana from the food box. Many of these solutions (e.g., roll/flip the box until the banana fell out or was visible near the hole) often required long handling times, whereas the no-manipulation reach-in technique allowed individuals to consistently retrieve the banana from the food box within a few seconds. Given that fast handling was key to obtaining the highly valued banana when dominant competitors were in the audience^55^, the perceived utility of learning the no-manipulation reach-in technique was likely closely tied to the risk of contest competition with dominant competitors. Because vervet monkeys form linear dominance hierarchies and live in highly despotic societies^53–56^, dominance rank would be expected to correlate closely with competition experienced. Indeed, we found this was the case when foraging as a group. Rank was a strong predictor of how likely individuals were to have a dominant competitor present or to be aggressed/displaced when group-foraging. However, adult and subadult males were more comfortable leaving the safety of the group and visiting the experiment alone. This allowed them to escape contest competition, such that they were less likely overall, to forage in the presence of dominant competitors. Notably, adult males experienced very little contest competition compared to the other age-sex classes. Not only were they comfortable foraging hundreds of meters away from the group for long periods, they were the highest-ranking group members throughout this study, and they rarely tried to participate in the experiment with another adult male. As a result, adult males rarely foraged in the presence of a dominant and were almost never displaced by a dominant when trying to retrieve the banana from the food box. For these reasons, adult males were likely less motivated than the other age-sex classes to learn an efficient foraging technique that mitigated the costs of contest competition.

Motivation is known to play an important role in learning in humans^22,23^, however, it is challenging to investigate the impact it has on learning in species that cannot self-report how motivated they felt to learn a given skill. When working with non-human animals, we must often infer motivation from an individual’s success, speed or persistence in learning the task^25–27,36,44,51,74^. In this study, we found that individuals that experienced more contest competition learned the no-manipulation reach-in technique faster. We also found that individuals that frequently experienced contest competition were more likely to attend to the handling techniques others used, particularly if they were still unskilled handlers themselves (i.e., had not learned the no-manipulation reach-in technique yet). These two observations suggest that unskilled monkeys were aware of their knowledge deficit (i.e. metacognition^75^), and if they frequently suffered from contest competition, they actively sought out social information they could exploit. A strategy that appears to have paid off, as the age-sex classes that were most attentive to social information were also the ones that showed improvement after they had opportunities to learn the no-manipulation reach-in technique from skilled demonstrators. Conversely, individuals who experienced very little contest competition were more likely to ignore valuable social information, fail to improve after opportunities for social learning, and instead learn inefficiently through individual learning. Thus, while we are forced to make inferences about how motivated individuals felt from their behaviour alone, we present a collection of findings that support that the monkeys were more motivated to learn a foraging skill if they were vulnerable to contest competition at food patches.

Our finding that adult males failed to exploit social information contrasts with a number of previous studies that have found adult males do engage in social learning^11,13,76^. One likely reason we did not observe more social learning among the adult males in this study is that the skill that was being learned (i.e., the no-manipulation reach-in technique) was an improvement over other techniques that were easy to learn and reliable provided access to the food reward. The initial goal of this experiment was to understand how competition impacted foraging decisions when food items varied in their handling time. Consequently, the food box was designed to increase handling time of the half banana, not to be a difficult task to solve. The monkeys did not need to lift or slide a door (solutions common in social learning experiments^13,76–78^) but could simply shake the box until the banana was visible through the hole and then grab it. This meant that adult males did not need to learn the no-manipulation reach-in technique to retrieve the food reward reliably. Once they had learned a technique that worked, albeit an inefficient one, they had relatively little motivation to improve their skills as they were not vulnerable to food loss to dominant competitors. In short, males were likely disinclined to seek out or adopt new possibilities (i.e., conservatism^79,80^). Furthermore, the longer that adult males stick with conservative strategies, the more difficult it may be to inhibit actions (e.g., rolling, tipping, or shaking the food box) that have previously provided them with access to a food reward^81^. A second reason that males may have been unlikely to engage in social learning in this study is that the social setting did not elicit conformity. In a well-known study, van de Waal and colleagues^11^ found that adult males that had recently immigrated into a new group conformed to the local social norms: they ignored their own knowledge about food palatability and began to eat the same food (i.e., color of corn) that the members of their new group preferred. In this experiment, the entire group would cluster around the two boxes of brightly-colored corn (i.e., there was a large audience) and because the rest of the group was feeding from one box, it would be conspicuous if the new male were to sit by himself at the other box. As such, it was easy for males to assess what the local social norm was, and their conformity to this norm was highly conspicuous to the members of their new group. Conversely, in our experiment, we only presented one food box at a time and trials were spaced out in time. This meant that there would be no conspicuous social norm to conform to. Additionally, the platforms could not be baited when the majority of the group was nearby as the monkeys would swarm the platforms and start to feed before baiting was complete. To avoid this, we waited until there were only a few monkeys around. This meant that the audience was usually small, or non-existent as adult males often visited the experiment site alone. As a result, there were typically few group members present to signal one’s conformity to.

Studies investigating individual variation in learning in wild animals must often use rough proxies for competitive ability (e.g., age, size, dominance rank) or hunger levels (e.g., fat scores, body weight) to test for motivational differences. However, when researchers fail to find an effect where one was expected, it is unclear if individual variation in motivation does not exist, or if the proxies used inadequately capture it. Here, we show that dominance rank does a poorer job of explaining individual variation in learning speed (and by inference, motivation to learn) than a measure that includes information on individual willingness to forage solitarily to escape contest competition. Dominance rank will likely perform even worse in species that are less social, live in less cohesive groups, have less despotic societies, or when resources are abundant in the environment or the food resource individuals are learning to exploit is less monopolizable (e.g., if multiple food boxes are presented to study subjects). All of these could result in lower levels of contest competition, and so would likely impact motivation to learn a new foraging technique. Therefore, we recommend that future studies endeavour to understand how individuals actually experience competition, and if appropriate, develop more accurate proxies for inferring motivation to learn.

Research interest in social learning and culture in non-human animals has surged over the past couple of decades, and with it, evidence for intra- and interindividual variation in the propensity to learn from others has also accrued^82^. Our findings highlight that motivation can be an important source of variation in learning efficiency and strategies (e.g., social versus individual). While the value of field studies in advancing our understanding of learning has already been lauded^83^, we argue that testing animals in wild settings is particularly critical to understanding the role that motivation plays in learning. Field studies allow researchers to test individuals in conditions that are socially and ecologically sound, where they are exposed to a broad range of challenges that could influence their motivation to access fitness-limiting resources. Conversely, it would be unethical to severely restrict food availability for captive subjects, or house them in social conditions that elicit the intense contest competition that can take place in the wild. Evidence for diminished motivation to learn in captivity is seen in studies that bring wild animals into the lab and observe motivation to decrease over time^31^, and the low participation rates that occur in captive studies, even when subjects have unrestricted access to the experimental apparatus^84^.

A fast-growing body of research highlights that a broad range of species acquire foraging, social and survival skills through social learning^11–20^. The spread of socially-learned skills has resulted in unique cultural traditions seen in many animals^85–87^. This breadth highlights the adaptive benefits of social learning. However, the work we present here highlights that even when individuals would benefit from using social information to improve their foraging skills, there can be significant variation among individuals in their motivation to use it. While quantifying motivation in non-human animals is challenging, a better understanding of the sources of individual variation in the perceived benefits of learning is key to growing our understanding of how traditions spread through populations.

## Supplementary Information

Supplementary Information 1.

Supplementary Information 2.

Supplementary Video 1.

Supplementary Video 2.

Supplementary Video 3.

Supplementary Video 4.

Supplementary Video 5.

Supplementary Video 6.

Supplementary Video 7.

Supplementary Video 8.

Supplementary Information 3.

Supplementary Information 4.

## Data Availability

The raw data supporting the conclusions of this article are available in the Electronic Supplementary Material.
