# Supplementary material for: Monkeys who experience more feeding competition utilize social information to learn foraging skills faster
Source: Sci Rep. 2023 Jul 19;13:11624. doi: 10.1038/s41598-023-37536-9 (PMC10356951; doi:10.1038/s41598-023-37536-9)
Supplement: Supplementary file 11 — Supplementary Information 3. [file 41598_2023_37536_MOESM11_ESM.docx]

**Electronic Supplementary Material**

**Monkeys who experience more feeding competition utilize social information to learn foraging skills faster**

**T. Jean M. Arseneau-Robar^1,2,3,*^ and Karyn A. Anderson^1,2,*^, Pascale Sicotte^3^ and Julie A. Teichroeb^1,2^**

**^1^ Department of Anthropology, University of Toronto Scarborough, Toronto, ON, Canada**

**^2^ Department of Anthropology, University of Toronto, Toronto, ON, Canada**

**^3^ Department of Biology, Concordia University, Montreal, QC, Canada**

**^*^ These two authors contributed equally to this paper**

**Corresponding author: T. Jean M. Arseneau-Robar**

**Email:** [**arseneau.jean.m@gmail.com**](mailto:arseneau.jean.m@gmail.com)

**ESM Video 1.** Vervet monkey manipulates food box until the banana falls out of the hole.

**ESM Video 2.** Vervet monkey manipulates food box until the banana is by the hole and can be retrieved by inserting only his hand into the box.

**ESM Video 3.** Vervet monkey exhibiting the no-manipulation reach-in technique.

**ESM Video 4.** Vervet monkey exhibiting the no-manipulation reach-in technique.

**ESM Video 5.** Audience member being attentive (i.e., looking for >1s) to the handling technique of a group member.

**ESM Video 6.** Audience member being attentive (i.e., looking for >1s) to the handling technique of a group member.

**ESM Video 7.** Audience member glancing (i.e., looking for <1s) at a competitor before/as trying to access another food platform.

**ESM Video 8.** Audience member glancing (i.e., looking for <1s) at a competitor before/as trying to access another food platform.


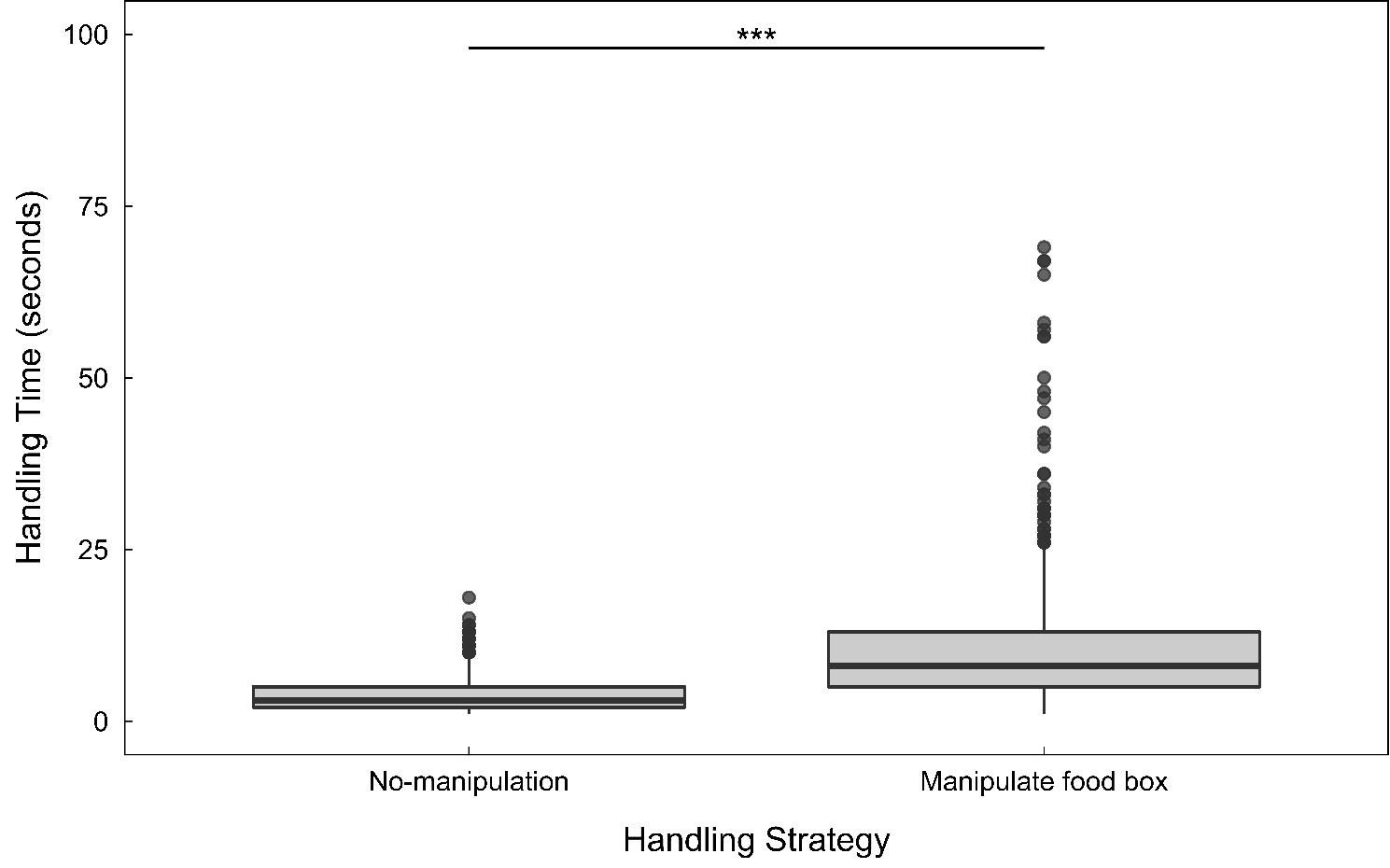


**ESM Figure 1.** Time it took monkeys to retrieve a food reward from a box when using handling techniques that involved manipulating the box (e.g., lifting, shaking, tipping, rolling, flipping), versus the no-manipulation reach-in technique.


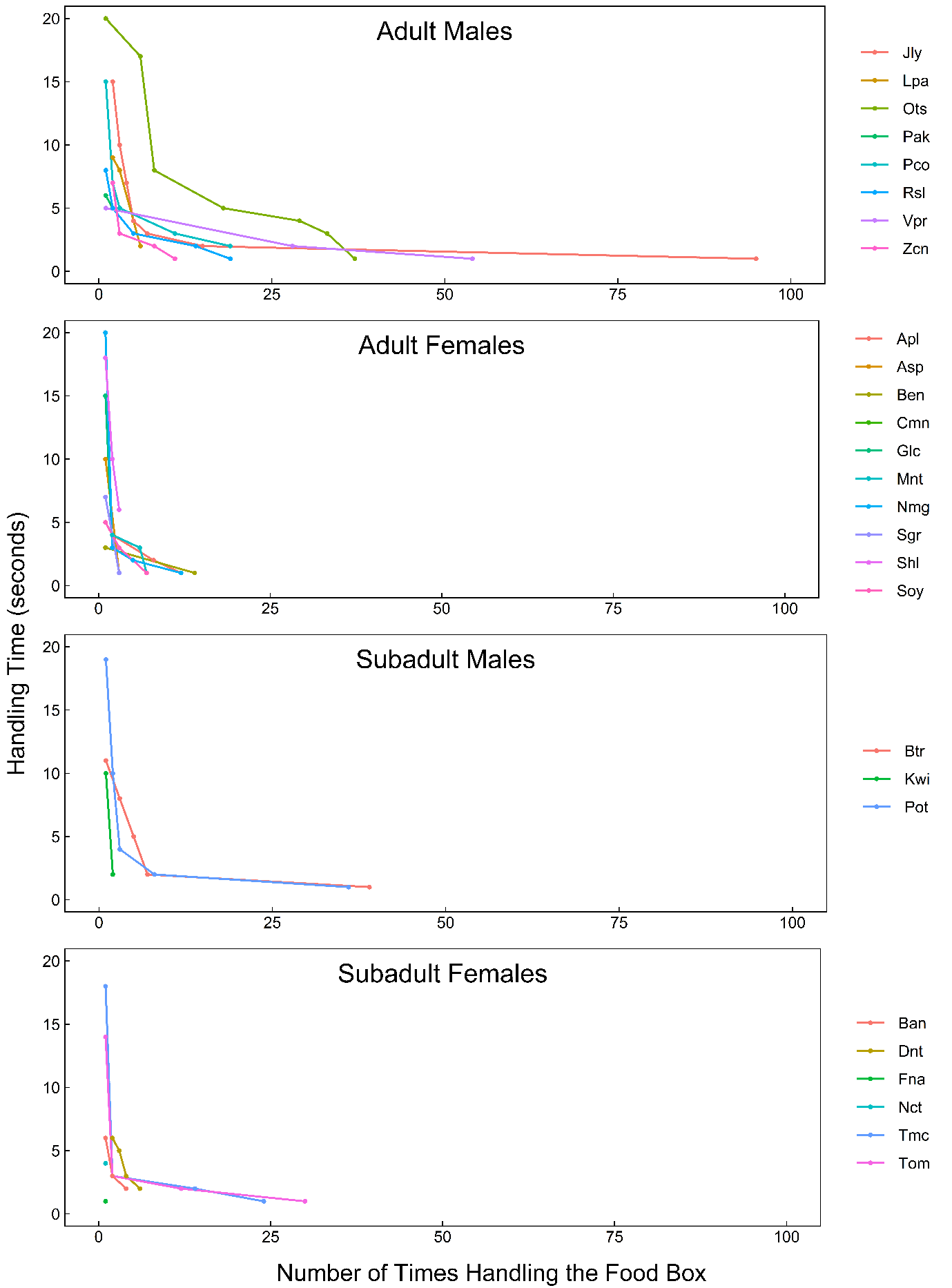


**ESM Figure 2.** Individuals within each age-sex class’s improvements in handling time with experience handling the food box. Only handling times that were lower than all previous handling times are presented; the right-most point in each individual’s improvement curve is their fastest handling time achieved.


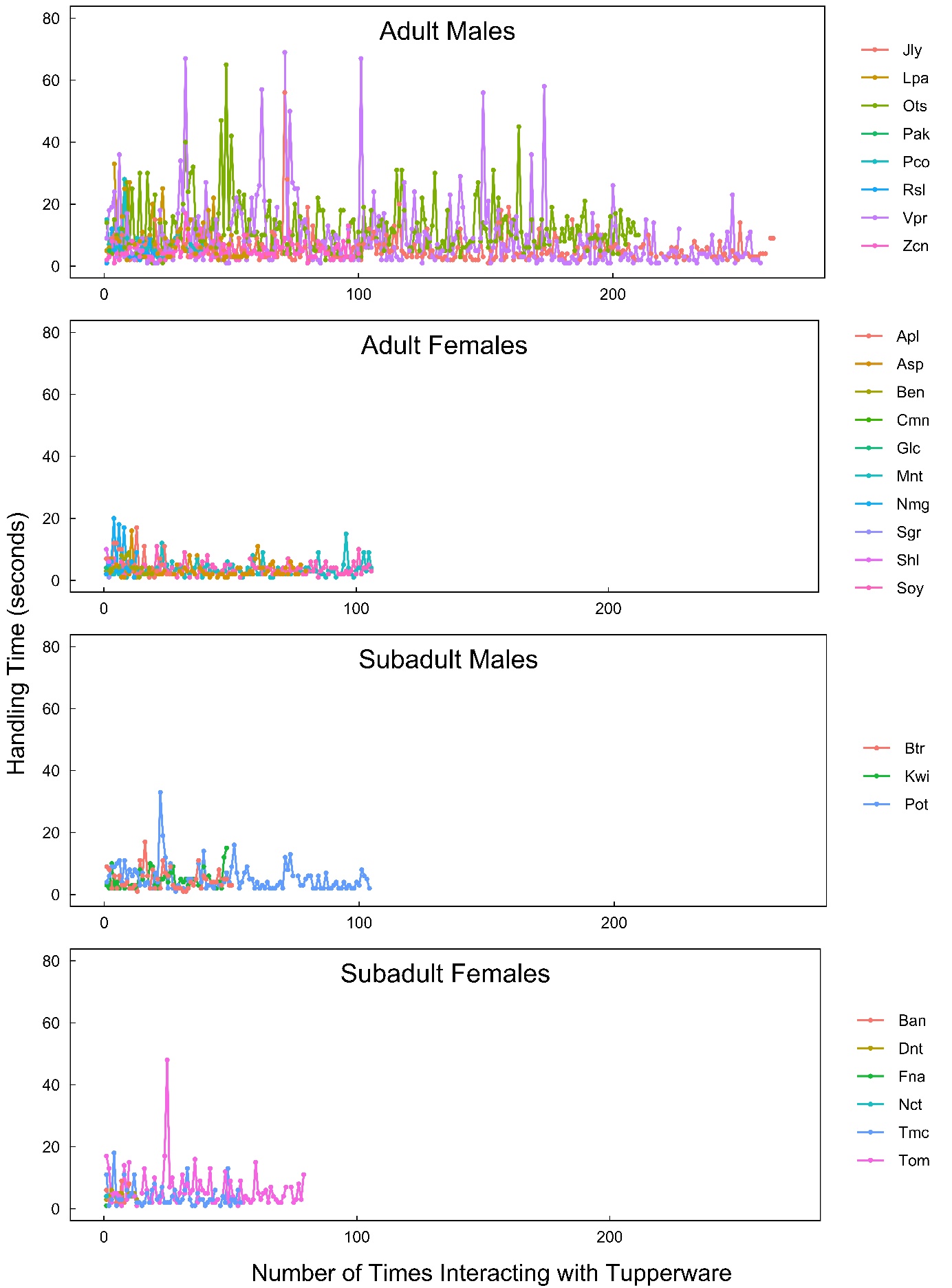


**ESM Figure 3.** Handling times for each study participant as they gained more experience handling the food box.

**ESM Table 1.** Sex, age class and rank (average Elo-score across study period) of each vervet monkey who participated in the study.

| Individual | Sex | Age-class | Rank (average Elo-score) |
| --- | --- | --- | --- |
| Jly | Male | Adult | 2339 |
| Pco | Male | Adult | 2290 |
| Ots | Male | Adult | 1973 |
| Rsl | Male | Adult | 1788 |
| Vpr | Male | Adult | 1743 |
| Lpa | Male | Adult | 1688 |
| Zcn | Male | Adult | 1545 |
| Pak | Male | Adult | 1543 |
| Mnt | Female | Adult | 1467 |
| Dnt | Female | Subadult | 1317 |
| Asp | Female | Adult | 1315 |
| Soy | Female | Adult | 1144 |
| Kwi | Male | Subadult | 1091 |
| Fna | Female | Subadult | 906 |
| Apl | Female | Adult | 889 |
| Ban | Female | Subadult | 879 |
| Sgr | Female | Adult | 799 |
| Pot | Male | Subadult | 745 |
| Ben | Female | Adult | 635 |
| Tom | Female | Subadult | 328 |
| Btr | Male | Subadult | 273 |
| Glc | Female | Adult | 248 |
| Cmn | Female | Adult | 161 |
| Nmg | Female | Adult | 118 |
| Tmc | Female | Subadult | -73 |
| Shl | Female | Adult | -156 |
| Nct | Female | Subadult | -362 |
